# Decomposition-Based Correlation Learning for Multi-Modal MRI-Based Classification of Neuropsychiatric Disorders

**DOI:** 10.3389/fnins.2022.832276

**Published:** 2022-05-25

**Authors:** Liangliang Liu, Jing Chang, Ying Wang, Gongbo Liang, Yu-Ping Wang, Hui Zhang

**Affiliations:** ^1^College of Information and Management Science, Henan Agricultural University, Zhengzhou, China; ^2^Department of Computer Science, Eastern Kentucky University, Richmond, KY, United States; ^3^Biomedical Engineering Department, Tulane University, New Orleans, LA, United States

**Keywords:** multi-modal, decomposition-based, matrix decomposition, canonical correlation analysis, neuropsychiatric disorders

## Abstract

Multi-modal magnetic resonance imaging (MRI) is widely used for diagnosing brain disease in clinical practice. However, the high-dimensionality of MRI images is challenging when training a convolution neural network. In addition, utilizing multiple MRI modalities jointly is even more challenging. We developed a method using decomposition-based correlation learning (DCL). To overcome the above challenges, we used a strategy to capture the complex relationship between structural MRI and functional MRI data. Under the guidance of matrix decomposition, DCL takes into account the spike magnitude of leading eigenvalues, the number of samples, and the dimensionality of the matrix. A canonical correlation analysis (CCA) was used to analyze the correlation and construct matrices. We evaluated DCL in the classification of multiple neuropsychiatric disorders listed in the Consortium for Neuropsychiatric Phenomics (CNP) dataset. In experiments, our method had a higher accuracy than several existing methods. Moreover, we found interesting feature connections from brain matrices based on DCL that can differentiate disease and normal cases and different subtypes of the disease. Furthermore, we extended experiments on a large sample size dataset and a small sample size dataset, compared with several other well-established methods that were designed for the multi neuropsychiatric disorder classification; our proposed method achieved state-of-the-art performance on all three datasets.

## 1. Introduction

Many neuropsychiatric disorders (NDs) not only result in a huge socioeconomic burden but are also accompanied by several comorbidities (Kessler et al., 2012). Although NDs arise from physical defects or injuries, they are usually considered a chronic course of mental disease, resulting in the collapse of an understanding of the real world, cognitive problems, and persistent damage (Heinrichs and Zakzanis, 1998). Diagnosis of NDs is important for tracking the development of the disease and for choosing and evaluating the effects of an intervention such as drug treatment. Furthermore, subtyping an ND can help in personalizing treatment. As a result, increasing attention has been paid to the identification of the subtypes of the ND, such as schizophrenia (SZ), bipolar disorder (BD), and attention deficit hyperactivity disorder (ADHD). However, it is difficult to distinguish these subtypes due to a lack of standard clinical criteria (McIntosh et al., 2005; Strasser et al., 2005; Finn et al., 2015; Liu Z. et al., 2018; Hu et al., 2019; Lake et al., 2019; Jiang et al., 2020).

Multi-modal magnetic resonance imaging (MRI) is a useful tool for clinical diagnosis of ND. It can provide information on different aspects of the brain. Functional MRI (fMRI) can be used to analyze the functional connections (FCs) between different brain regions. These FCs reveal individual differences in neural activity patterns, which can predict continuous phenotypic measurements (Dubois and Adolphs, 2016; Rosenberg et al., 2018; Hu et al., 2021). On the other hand, structural MRI (sMRI) reflects the location, volume, and lesions of brain tissue (McIntosh et al., 2005; Liu et al., 2019), in addition to providing information about structural connections among brain regions (Wang et al., 2009). A number of MRI studies have been conducted on ND classification, including Alzheimer's disease (Fan et al., 2020), ADHD (Connaughton et al., 2022), SZ (de Filippis et al., 2019), BD (Madeira et al., 2020), depression (Han et al., 2019), and autism (Rakić et al., 2020). However, most of these studies focus only on one type of MRI image or one type of ND. They overlook complementary information, resulting in lower classification accuracy.

Compared to natural image studies, the limited number of medical MRI samples is a challenge for the state-of-the-art convolutional neural networks and graph convolutional networks (Yu et al., 2019; Willemink et al., 2020). In particular, the high-dimensionality of MRI and nonlinear relations between the matrices of MRIs pose challenges for these machine learning methods. In addition, the imaging principles of sMRI and fMRI are different, and there is no direct correlation between them. Exploring the relationship between them is itself challenging.

Previous multi-modal MRI studies have demonstrated the potential of a multi-modal fusion approach in studying the relationship between fMRI and sMRI images (Qiao et al., 2019; Gao et al., 2020; Jiang et al., 2021; Mill et al., 2021). For example, Qiao et al. (2019) proposed a hybrid feature selection method based on statistical approaches and machine learning. This method explored the brain abnormalities in SZ using both fMRI and sMRI images. A multi-kernel support vector machine (SVM) was used for SZ classification, which was based on the similarity of the decomposed components from multi-modal MRI (Gao et al., 2020). Jiang et al. (2021) combined the multi-dimensional features of sMRI and fMRI to predict the state of SZ and guide medication. Different modalities contain complementary information, which can improve the performance of the model (Jiang et al., 2021; Mill et al., 2021). However, the poor interpretability of some models has become an issue when identifying significant biomarkers (Olesen et al., 2003; Seghier et al., 2004). Various strategies are widely used in multi-modal data analysis, including multi-modal canonical correlation analysis (CCA) (Correa et al., 2010), deep collaborative learning (Hu et al., 2019), parallel independent component analysis (Liu et al., 2008), and methods similar to independent component analysis (Sui et al., 2009; Calhoun et al., 2010; Groves et al., 2011).

Some previous studies have identified a correlation between fMRI and sMRI images in ND groups (Sui et al., 2011; Qiao et al., 2019; Su et al., 2020). Therefore, we propose a prediction method, called decomposition-based correlation learning (DCL), for the multi-modal MRI-based classification of NDs. We first used the shrinkage principal orthogonal complement thresholding method (S-POET) (Fan and Wang, 2015) to estimate spiked fMRI and sMRI matrices. Subsequently, in the DCL method, we use decomposition-based CCA to decompose each pair of matrices into two common matrices and two orthogonal distinctive matrices. Finally, we computed the correlation between the common matrices and the distinctive matrices. We validated the DCL method on the Consortium for Neuropsychiatric Phenomics (CNP) dataset. Our results demonstrate that the proposed DCL model outperforms several other methods. We also discovered interesting feature connections when identifying significant features in fMRI data.

The rest of this paper is organized as follows. Section 2 describes the DCL pipeline and provides a quantitative evaluation of our method. The dataset and experiments in applying DCL to NDs are presented in Sections 3, 4. A discussion and analysis of the results are in Section 5. Section 6 concludes this paper.

## 2. Methodology

**Figure 1:**
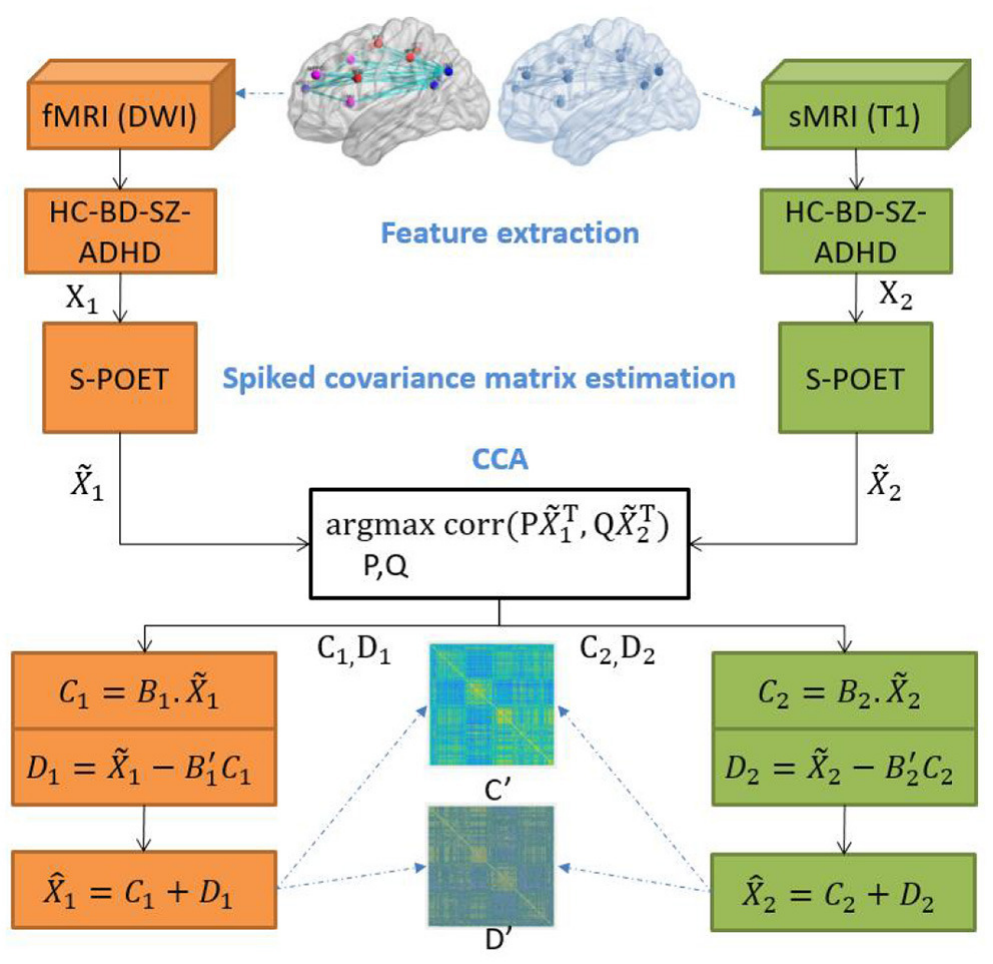
Overview of the architecture of the proposed integration model.

The DCL pipeline is shown in Figure 1. DCL has three steps: data processing (feature extraction), S-POET (spiked covariance matrix estimation), and CCA (canonical correlation and matrix construction).

### 2.1. Overview of Principal Component Analysis (PCA)

Principal component analysis is a powerful tool for feature extraction and data visualization. PCA can extract principal components from multivariate data by maximizing the variance of the features while minimizing the reconstruction error.

Let *X* ∈ ℝ^*m*×*n*^ be a matrix, where *m* and *n* are the size of the matrix. Hence,


(1) $$\begin{array}{l} X=\left[x_{1},x_{2},x_{3},\ldots ,x_{m}\right]. \end{array}$$

Let $\hat{X}$ be the average signal, which is defined as follows:


(2) $$\begin{array}{l} \hat{X}=\frac{1}{m}\overset{m}{\underset{n=1}{\sum }}x_{n}. \end{array}$$

The normalized vectors are computed by subtracting the average signal from each training vector. They are defined as follows:


(3) $$\begin{array}{l} \phi_{i}=x_{i}-\hat{X}. \end{array}$$

These vectors go through PCA. Let *C* be a covariance matrix:


(4) $$\begin{array}{l} C=\frac{1}{m}\overset{m}{\underset{n=1}{\sum }}\phi_{n}\phi_{i}^{⊤}. \end{array}$$

### 2.2. Overview of S-POET

The shrinkage principal orthogonal complement thresholding method (Fan and Wang, 2015) is a covariance estimator with an approximate factor model. It is based on sparse PCA. Feature matrices from fMRI and sMRI data are input into S-POET, which calculates an asymptotic first-order distribution for the eigenvalues and eigenvectors of the sample correlation matrices.

Specifically, let *k* be the number of datasets and *n* be the number of samples in the *k*-th dataset. A high-dimensional dataset can be written as matrix $\tilde{X}\in {\mathbb{R}}^{p_{k}\times n}$. In our experiment, we have two matrices, one from fMRI and one from sMRI, so we set *k* = 2. *p*_*k*_ is a row, which corresponds to a mean-zero variable. S-POET constructs ${\tilde{X}}_{k}$, which is the estimate of matrix *X*_*k*_. Before defining ${\tilde{X}}_{k}$, we let the full singular value decomposition of *Y*_*k*_ be as follows:


(5) $$\begin{array}{l} Y_{k}=V_{k1}\lambda_{yk}V_{k2}^{⊤}, \end{array}$$

where *V*_*k*1_ and *V*_*k*2_ are two orthogonal matrices. λ_*yk*_ is a rectangular diagonal matrix whose singular values on the main diagonal are arranged in descending order. ${\tilde{X}}_{k}$ is a matrix:


(6) $$\begin{array}{l} {\tilde{X}}_{k}=V_{k1}^{\left[:,1:r_{k}\right]}\mathrm{diag}\left({\hat{\sigma }}_{1}^{S}\left(Y_{k}\right),\ldots ,{\hat{\sigma }}_{r_{k}}^{S}\left(Y_{k}\right)\right)(V_{k2}^{\left[:,1:r_{k}\right]}{)}^{⊤}, \end{array}$$

(7) $$\begin{array}{l} {\hat{\sigma }}_{l}^{S}\left(Y_{k}\right)=\sqrt{\max\left\{\sigma_{l}^{2}\left(Y_{k}\right)-\tau_{k}p_{k},0\right\}}, \end{array}$$

(8) $$\begin{array}{l} \tau_{k}=\overset{p_{k}}{\underset{l=r_{k}+1}{\sum }}\sigma_{l}^{2}\left(Y_{k}\right)/\left(np_{k}-nr_{k}-p_{k}r_{k}\right), \end{array}$$

where ${\tilde{r}}_{k}=\mathrm{rank}\left({\tilde{X}}_{k}\right)$ and ${\tilde{r}}_{k}=r_{k}$.

We summarize the S-POET method in Algorithm 1.

**Algorithm 1:** S-POET

**Input:** $\text{X}\in {\mathbb{R}}^{p_{k}\times n}$ **Output:** ${\tilde{\text{X}}}_{k}$
1: *K* ← rank cov(***X***) //*Covariance estimator*
2: *p, n* ← shape(***X***)
3: *V, S, U*_*t*_ ← SVD(***X***, *full*_*matrices*_ = *False*)
4: *S* ← diag(*S*)
5: *Lambda* ← *S* **2/*n* //*lambda expression*
6: $\tilde{c}\leftarrow Sum\left(Lambda.diagonal()\left[K:\right]\right)/\left(p-K-p*K/n\right)$
7: $Lambda_{s}\leftarrow Maximum\left(Lambda\left[:K,:K\right]-\tilde{c}*p/n,0\right)$
8: ${\tilde{\text{X}}}_{k}\leftarrow V\left[:,:K\right]@\mathrm{Sqrt}\left(Lambda_{s}*n\right)@U_{t}\left[:K,:\right]$
9: **return** ${\tilde{\text{X}}}_{k}$, *Lambda*_*s*_, *V*[:, :*K*], K

### 2.3. Overview of CCA

Canonical correlation analysis is a multivariate statistical analysis method. It determines the overall correlation between two groups of indicators. We use CCA to examine the cross-covariances of multi-modal MRI data.

Let ${\tilde{X}}_{1}\in {\mathbb{R}}^{n\times r}$ and ${\tilde{X}}_{2}\in {\mathbb{R}}^{n\times s}$ be two matrices, where *n* is the number of samples, and *r* and *s* are the feature sizes of the two matrices, respectively. CCA is used to find two coefficient vectors $v_{1}\in {\mathbb{R}}^{r\times 1}$ and $v_{2}\in {\mathbb{R}}^{s\times 1}$ by optimizing the Pearson correlation between ${\tilde{X}}_{1}v_{1}$ and ${\tilde{X}}_{2}v_{2}$, which is defined as follows:


(9) $$\begin{array}{l} \left(v_{1}^{*},v_{2}^{*}\right)=\underset{v_{1},v_{2}}{\arg\max}v_{1}^{\prime }\Phi_{12}v_{2}, \end{array}$$

where $v_{1}^{\prime }\Phi_{11}v_{1}=1$, $v_{2}^{\prime }\Phi_{22}v_{2}=1$, $v_{1}\in {\mathbb{R}}^{r\times 1}$, $v_{2}\in {\mathbb{R}}^{s\times 1}$, and $\Phi_{ij}={\tilde{X}}_{i}^{\prime }{\tilde{X}}_{j}$. ${\tilde{X}}_{1}v_{1}$ and ${\tilde{X}}_{2}v_{2}$ are two identified canonical vectors, both of which are linear combinations of raw features in the original data, ${\tilde{X}}_{1}$ and ${\tilde{X}}_{2}$, respectively. ${\tilde{X}}_{1}v_{1}$ and ${\tilde{X}}_{2}v_{2}$ facilitate the interpretation of multi-omics associations by reducing the dimensionality (${\tilde{X}}_{1}v_{1},{\tilde{X}}_{2}v_{2}\in {\mathbb{R}}^{n\times 1}$). We use Equation (9) as a constraint, and $v_{1}^{\prime }\Phi_{12}v_{2}$ can be used as the cross-data correlation, i.e.,


$$v_{1}^{\prime }\Phi_{12}v_{2}=\frac{v_{1}^{\prime }\Phi_{12}v_{2}}{\sqrt{v_{1}^{\prime }\Phi_{11}v_{1}v_{2}^{\prime }\Phi_{22}v_{2}}}.$$

Canonical correlation analysis is used to guarantee the highest total correlation of the pair-wise independent canonical vectors, which is defined as follows:


(10) $$\begin{array}{l} \left(V_{1}^{*},V_{2}^{*}\right)=\underset{V_{1},V_{2}}{\arg\max}\mathrm{trace}\left(V_{1}^{\prime }\Phi_{12}V_{2}\right), \end{array}$$

where $V_{1}^{\prime }\Phi_{11}V_{1}=V_{2}^{\prime }\Phi_{22}V_{2}=I_{n}$, $V_{1}\in {\mathbb{R}}^{r\times k}$, $V_{2}\in {\mathbb{R}}^{s\times k}$, and $k=\text{min}\left[\text{rank}\left({\tilde{X}}_{1}\right),\text{rank}\left({\tilde{X}}_{2}\right)\right]$. Since Φ_11_ and Φ_22_ may be singular when calculating the loading vectors, matrix regularization is usually enforced on them to ensure that they are positive definite:


(11) $$\begin{array}{l} \begin{array}{c} \hat{\Phi_{11}}=\Phi_{11}+r_{1}I_{r},\\ \hat{\Phi_{22}}=\Phi_{22}+r_{2}I_{r}. \end{array} \end{array}$$

### 2.4. Decomposition-Based Correlation Learning

Let *X*_1_ and *X*_2_ be paired matrices of fMRI and sMRI, which are the input of S-POET methods. We use the DCL method to decompose this pair of matrices into two common matrices and two orthogonal distinctive matrices. Then, we collect these two types of matrices into a common matrix (*C*_*k*_) and a distinctive matrix (*D*_*k*_), respectively. Based on the output (${\tilde{X}}_{k}$) of S-POET, we use ${\tilde{X}}_{k}$ to develop two estimators for *C*_*k*_ and *D*_*k*_. First, we define the common variable *c*_*base*_ as follows:


(12) $$\begin{array}{l} c_{base}\propto n^{-1}\underset{w\in \left(l_{0}^{2},cov\right)}{\arg\max}\left\{{\mathrm{corr}}^{2}\left(X_{1},w\right)+{\mathrm{corr}}^{2}\left(X_{2},w\right)\right\}, \end{array}$$

where the constraints *X*_1_ = *C*_1_ + *D*_1_, *X*_2_ = *C*_2_ + *D*_2_, corr(*D*_1_, *D*_2_) = 0, and *c*_*base*_ ∈ [0, 1].

Then, the estimator of *C*_*k*_ can be defined as follows:


(13) $$\begin{array}{l} {\hat{C}}_{k}=n^{-1}{\tilde{X}}_{k}({\hat{V}}_{k}^{\left[1:r_{12},:\right]}{)}^{⊤}{\hat{A}}_{C}^{\left(r_{12}\right)}\overset{2}{\underset{j=1}{\sum }}{\hat{V}}_{k}^{\left[1:r_{12},:\right]}c_{base}, \end{array}$$

where ${\hat{A}}_{C}^{\left(r\right)}=\text{diag}\left({\hat{a}}_{1},\ldots {\hat{a}}_{r}\right)$, *C*_1_ and *C*_2_ have the maximum correlation between each other, while the vectors within each are uncorrelated and whitened. Their correlation vectors ${\hat{a}}_{1}$, ${\hat{a}}_{2}$,…, ${\hat{a}}_{r}$ are called the canonical correlation coefficients.

The estimator of *D*_*k*_ is defined as follows:


(14) $$\begin{array}{l} {\hat{D}}_{k}={\tilde{X}}_{k}-n^{-1}{\tilde{X}}_{k}({\hat{V}}_{k}^{\left[1:{\tilde{r}}_{12},:\right]}{)}^{⊤}{\hat{A}}_{C}^{\left({\tilde{r}}_{12}\right)}\overset{2}{\underset{j=1}{\sum }}{\hat{V}}_{k}^{\left[1:{\tilde{r}}_{12},:\right]}c_{base}. \end{array}$$

In our experiment, we use the relationship between ${\hat{D}}_{1}$ and ${\hat{D}}_{2}$ to represent the orthogonal relationship between two distinctive matrices, and ${\hat{D}}_{1}{\hat{D}}_{2}=0_{p1\times p2}$. Finally, ${\hat{X}}_{k}$, the estimator of *X*_*k*_, is defined as follows:


(15) $$\begin{array}{l} {\hat{X}}_{k}={\hat{C}}_{k}+{\hat{D}}_{k}. \end{array}$$

We summarize DCL in Algorithm 2.

**Algorithm 2:** DCL

**Input:** ${\text{X}}_{1}\in {\mathbb{R}}^{p\times n}$, ${\text{X}}_{2}\in {\mathbb{R}}^{s\times n}$ //Input of sMRI and fMRI, respectively. **Output:** ${\hat{\text{X}}}_{1}$,${\hat{\text{X}}}_{2}$
1: ${\tilde{\text{X}}}_{1},Lambda_{1},U_{1}\leftarrow S-POET\left({\text{X}}_{1}\right)$ //processed by S-POET method
2: ${\tilde{\text{X}}}_{2},Lambda_{2},U_{2}\leftarrow S-POET\left({\text{X}}_{2}\right)$ //processed by S-POET method
3: *Lambda*_11_ ← Construct diag(*Lambda*_1_)
4: *Lambda*_22_ ← Construct diag(*Lambda*_2_)
5: $Theta\leftarrow \left(Lambda_{11}@U_{1}.T@{\tilde{\text{X}}}_{1}\right)@\left({\tilde{\text{X}}}_{2}.T@U_{2}@Lambda_{22}\right)/n$
6: *V*_*theta*_, *D*_*theta*_ ← SVD(*Theta, full*_*matrices*_ = *True*) //Singular Value Decomposition
7: *Gamma*_1_ ← *U*_1_@*Lambda*_11_@*V*_*theta*_
8: *Gamma*_2_ ← *U*_2_@*Lambda*_22_@*V*_*theta*_
9: ***A***_*mat*_ ← diag(*D*_*theta*_) //Diagonal matrix
10: ***C***_*base*_ ← Common variables $\mathrm{corr}\left({\tilde{\text{X}}}_{1},{\tilde{\text{X}}}_{2}\right)$
11: ${\tilde{\text{C}}}_{1}\leftarrow$ Common matrix $\left({\tilde{\text{X}}}_{1},C_{base},A_{mat}\right)$
12: ${\tilde{\text{C}}}_{2}\leftarrow$ Common matrix $\left({\tilde{\text{X}}}_{2},C_{base},A_{mat}\right)$
13: ${\tilde{\text{D}}}_{1}\leftarrow$ Distinctive matrix $\left({\tilde{\text{X}}}_{1},{\tilde{C}}_{1}\right)$
14: ${\tilde{\text{D}}}_{2}\leftarrow$ Distinctive matrix $\left({\tilde{\text{X}}}_{2},{\tilde{C}}_{2}\right)$
15: ${\tilde{\text{X}}}_{1}\leftarrow$ Combination of common and distinctive matrices
16: ${\tilde{\text{X}}}_{2}\leftarrow$ Combination of common and distinctive matrices
17: **return** ${\hat{\text{X}}}_{1}$, ${\hat{\text{X}}}_{2}$

## 3. Methods

### 3.1. CNP Dataset

We evaluated the proposed DCL method in classifying NDs in the CNP dataset (Poldrack et al., 2016). The CNP dataset was collected by a consortium at the University of California, Los Angeles (UCLA), with financial support provided by the National Institutes of Health. This dataset has been used to elucidate the association between the human genome and complex psychological syndromes and promote the development of new therapies for NDs. All of this research was based on image phenotypic features in the mental disease.

The consortium for neuropsychiatric dataset was obtained from the OpenfMRI project (Gorgolewski et al., 2016). It includes sMRI data, task-based fMRI data, and resting-state fMRI data. These MRI images were acquired on one of two 3T Siemens Trio scanners at UCLA. The database contains extensive details of neuropsychologic assessments, neurocognitive tasks, and demographic information (including biological sex, age, and education). In addition, there are also details of the medication taken by those in ND groups.

The present study includes 272 images of subjects in one of four categories: 130 healthy controls (HCs), 50 SZ subjects, 49 BD subjects, and 43 ADHD subjects. These 272 images were from people in the Los Angeles area aged between 21 and 50 years old who were recruited through community advertisements. The details of the CNP dataset are listed in Table 1.

**Table 1:** Details of the Consortium for Neuropsychiatric Phenomics (CNP) dataset.

**ID**	**Subtype**	**Number**	**Details**
0	Healthy controls (HC)	130	–
1	Schizophrenia (SZ)	50	Disorganized, paranoid, or residual types
2	Bipolar disorder (BD)	49	Most recent hypomanic or manic episode, mild or moderate
3	Attention deficit hyperactivity disorder (ADHD)	43	Predominantly inattentive, combined, or predominantly hyperactive-impulsive types

### 3.2. Brain Connectivity Data

Brain connectivity information may be reflected in fMRI images. In the CNP dataset, each sample has seven fMRI modalities, which were collected during different task states: BOLD contrast, resting state (with physiological monitoring), breath-holding tasks (with physiological monitoring), balloon analog risk tasks, stop-signal tasks, task switching, and spatial working memory capacity tasks. In this study, we attempted to classify NDs using resting-state fMRI images.

Resting-state fMRI is an imaging technique that obtains a brain activity function map when the subject is in a resting state undisturbed by other activities, which is better for distinguishing ND groups. The CNP dataset has resting-state fMRI images with scans lasting 304 s. The participants were relaxed with their eyes open. They were not stimulated or asked to respond during scanning (Poldrack et al., 2016). The fMRI data were collected under the following parameters: the slice thickness was 4 mm, 34 slices were taken, TR was 2 s, TE was 30 ms, the flip angle was 90°, the matrix size was 64 × 64, the field of view was 192 mm, and the orientation was an oblique slice. In addition, high-resolution anatomical MP-RAGE data were collected under the following parameters: TR was 1.9 s, TE was 2.26 ms, the field of view was 250 mm, the matrix size was 256 × 256, the slices were in the sagittal plane, the slice thickness was 1 mm, and 176 slices were taken. We excluded 24 samples for which the whole-brain image volumes were unavailable or the head had moved excessively. Finally, we had 248 samples.

Before subsequent experiments, we preprocessed the fMRI data according to Gorgolewski et al. (2017), including slice timing, head motion corrections, spatial smoothing, band-pass filtering (0.01–0.1 Hz), nuisance signal regression, and Montreal Neurological Institute (MNI) space normalization and so on. Then, we used FSL to skull stripped and co-registered fMRI to the corresponding T1 weighted volume using boundary based registration with 9 degrees of freedom implemented in FreeSurfer. Finally, we obtained the functional connectivity matrix of the brain through the following steps: first, we used the BioImage Suite (Joshi et al., 2011) to calculate connectivity matrices for the fMRI images. We then used the Anatomical Automatic Labeling 90 (AAL90) brain atlas, which divided the brain images into 90 regions. The Pearson correlation coefficient was used to calculate the node values. The Fisher transformation was used to normalize the *z* scores. Finally, we obtained a 90 × 90 symmetric connectivity matrix for each sample. These connectivity matrices were not thresholded or binarized.

### 3.3. Brain Structure Data

Structural MRI are also used as inputs to the DCL method. It was obtained with the same parameter values used for the fMRI images. We used the open-source software FreeSurfer to process and analyze these sMRI images. FreeSurfer is used to analyze and visualize cross-sectional structural images. It can be used for stripping the skull, correcting the B1 bias field, registering an image, reconstructing the cortical surface, and estimating the cortical thickness.

We used FreeSurfer to generate high-precision gray and white matter segmentation surfaces and gray matter and cerebrospinal fluid segmentation surfaces. From these two surfaces, we calculated the cortical thickness and other surface features, such as the cortical surface area, curvature, and gray matter volume. Overall, there were 248 subjects, we obtained 2,196 features from the sMRI image of a subject. Finally, we constructed a 248 × 2, 196 matrix from the sMRI image of 248 subjects.

## 4. Experiments and Results

### 4.1. Experimental Design and Metrics

In our experiments, we focused on two aspects of brain connectivity: (1) classifying NDs into different subtypes using fMRI and sMRI data and (2) extracting important features from the fMRI and sMRI images. The classification task was to validate the performance of the DCL method for the different ND groups, whereas the feature extraction task was used to assess the capability of the method in detecting correlated features.

We obtained the correlation matrices by inputting the 248 fMRI (90 × 90) and sMRI (248 × 2196) matrices into S-POET. Then, we decomposed each pair of canonical matrices and computed their correlations. Finally, we used the leave-one-out (LOO) method to select the important features in the test sample matrix. For a dataset with *n* samples, verification based on LOO is carried out over *n* iterations. In each iteration, the classifier uses *n* − 1 samples as training samples and uses the remaining sample as testing samples.

In our experiments, accuracy (ACC), precision (PRE), recall (REC), and F-score (*F*_1_) are used to measure the classification performance. They are defined as follows:


(16) $$\begin{array}{l} \text{ACC}=\frac{\text{TP }+\text{ TN}}{\text{TP }+\text{ TN }+\text{ FP }+\text{ FN}},\\ \text{PRE}=\frac{\text{TP}}{\text{TP }+\text{ FP}},\\ \text{REC}=\frac{\text{TP}}{\text{TP }+\text{ FN}},\\ \;F_{1}=2\frac{\text{PRE }\times \text{ REC}}{\text{PRE }+\text{ REC}}, \end{array}$$

where TP is the number of true positives, TN is the number of true negatives, FP is the number of false positives, and FN is the number of false negatives. The values of these metrics were obtained from a LOO-based cross-validation.

Our experiments were implemented in Python on an NVIDIA Titan X Pascal CUDA GPU processor.

### 4.2. LOO Classification Method

We compared the performance of the DCL method with other methods: SVM, random forest (RF), XGBoost, PCA+SVM, PCA+RF, PCA+XGBoost, CCA+SVM, CCA+RF, and CCA+XGBoost. The linear kernel in the SVM classifier was used, as it provides better experimental performance than other kernels. As a trade-off between performance and computational cost, we set the number of trees in RF to 100. To prevent overfitting by XGBoost, we set the maximum tree depth for base learners and the turning parameter for the *L*_2_ regularization term to 10 and 5, respectively. In the experiments, SVM, RF, and XGBoost use concatenated fMRI and sMRI matrices as their input, while the fMRI and sMRI matrices input to the other methods were first processed by the PCA, CCA, or DCL methods.

The classification results for the DCL method and the other classifiers are shown in Table 2. Each experiment was verified with 10-fold cross-validation. The conventional machine learning classifiers (SVM, RF, and XGBoost) had the lowest accuracy. These classifiers cannot capture distinguishable information from the union matrix. Compared with SVM, RF, and XGBoost, the PCA and CCA classifiers achieved better classification results. The best accuracy for both was 49.00%, which demonstrates that correlation information can be incorporated to improve the classification. The classifiers based on DCL had much better performance than those based on PCA or CCA. The best accuracy was 72.00%. Our proposed DCL method is a natural extension of the traditional CCA method. Based on the CCA decomposition, DCL determines the common and discernibility matrices and establishes an orthogonal relationship between the two discernibility matrices.

**Table 2:** Mean values in the evaluation of the classification performance on the CNP dataset.

**Classifier**	**ACC (%)**	**PRE (%)**	**REC (%)**	***F*_1_ (%)**
SVM	38.00 (4.00)	40.00 (10.00)	39.00 (5.00)	37.00 (6.00)
RF	41.00 (10.00)	32.00 (11.00)	42.00 (7.00)	35.00 (9.00)
XGBoost	45.00 (6.00)	32.00 (9.00)	46.00 (4.00)	36.00 (5.00)
PCA+SVM	46.00 (2.00)	43.00 (7.00)	50.00 (7.00)	40.00 (3.00)
PCA+RF	47.00 (9.00)	49.00 (7.00)	46.00 (6.00)	44.00 (3.00)
PCA+XGBoost	49.00 (11.00)	45.00 (8.00)	49.00 (8.00)	45.00 (7.00)
CCA+SVM	45.00 (9.00)	42.00 (18.00)	49.00 (15.00)	38.00 (12.00)
CCA+RF	47.00 (13.00)	48.00 (11.00)	48.00 (10.00)	43.00 (10.00)
CCA+XGBoost	49.00 (8.00)	46.00 (14.00)	49.00 (12.00)	44.00 (14.00)
DCL+SVM	64.00 (9.00)	69.00 (7.00)	66.00 (6.00)	65.00 (8.00)
DCL+RF	68.00 (10.00)	73.00 (3.00)	72.00 (4.00)	72.00 (4.00)
DCL+XGBoost	72.00 (8.00)	81.00 (2.00)	70.00 (3.00)	75.00 (3.00)

In addition, our comparative experiment was based on a sample size of 248. As shown in Table 2, we used three typical machine learning methods (SVM, RF, and XGBoost) as the baseline. The performance of these three machine learning methods was very different from that based on the PCA, CCA, or DCL methods. There are two reasons:

1. Machine learning methods can be effective for classifying simple images, but because medical images are very complex, these three machine learning methods were overwhelmed.
2. The limited sample size does not meet the training requirements of the three machine learning methods. The multi-class classification task increased the imbalance for the samples, making it difficult for these methods to obtain key feature information from the high latitude and limited samples.

Therefore, unlike the other methods, the DCL method first preprocesses the complex relationship between the sMRI and fMRI data, which reduces the complexity of the input data. Table 2 shows that, despite the limited sample size, DCL can better deal with the relations in high latitude data and improve the performance of machine learning.

**Figure 2:**
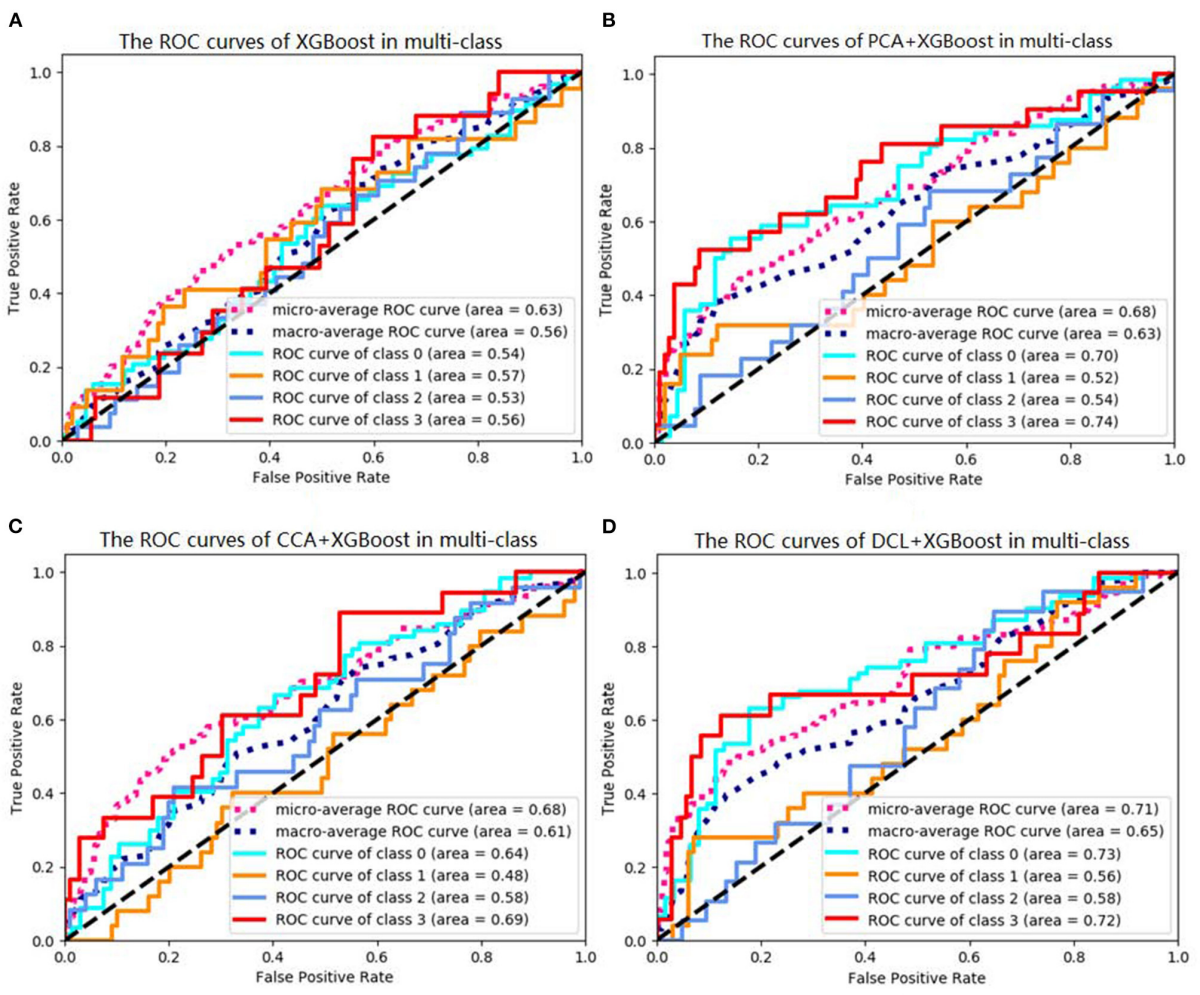
Receiver operating characteristics (ROC) curves of XGBoosts with different pretreatment methods. **(A)** XGBoost method is used in classification task. **(B)** The PCA-based XGBoost is used in classification task. **(C,D)** CCA and DCL-based XGBoosts are used in classification task.

Of the DCL-based classifiers, XGBoost had the best results in the multi-class classification task. The best accuracy was 72.00%. The receiver operating characteristic (ROC) curves for XGBoost in multi-class classification is plotted in Figure 2. The areas under the micro-averaged and macro-averaged ROC curves in Figures 2B,C are much larger than those in Figure 2A. Moreover, the areas under the curves for the four subtypes in Figures 2B,C are much larger than those in Figure 2A. These results indicate that the correlation information obtained by PCA or CCA can improve the performance of a classifier. The classification results for DCL are much better than those for PCA or CCA. The areas under all the ROC curves in Figure 2D are larger than those in Figures 2B,C. This indicates that our DCL method can better describe brain connection networks and thus improve the performance of the classifiers.

### 4.3. Feature Selection Based on the LOO Method

Besides assessing the performance of the DCL method, we also identified the important features with the DCL+XGBoost method. The aim was to find which edges contribute to brain connectivity. The extracted features are mapped back into the brain space, which facilitates the interpretation of the known relationship between brain structure and function. However, due to the dimensionality of the connectivity network, the visualization is challenging. In the LOO method, we used a weight-based method to evaluate the importance of features in the test sample matrix. The weight in XGBoost is used to calculate the number of times a feature is used as a split point across all trees. Finally, we counted the number of samples whose feature weights were >0. We visualized the representations of all important features for both the sMRI and fMRI data.

### 4.4. Visualization of FCs

It is interesting to investigate how different brain networks cooperate and connect with each other. We found that there were significant differences between the FCs of each group, which indicates that these FCs not only reflect the information common to the different groups but also the differences among them. We used the BrainnetViewer software (https://www.nitrc.org/projects/bnv/) to visualize which FCs have the strongest relationships in the brain network.

**Figure 3:**
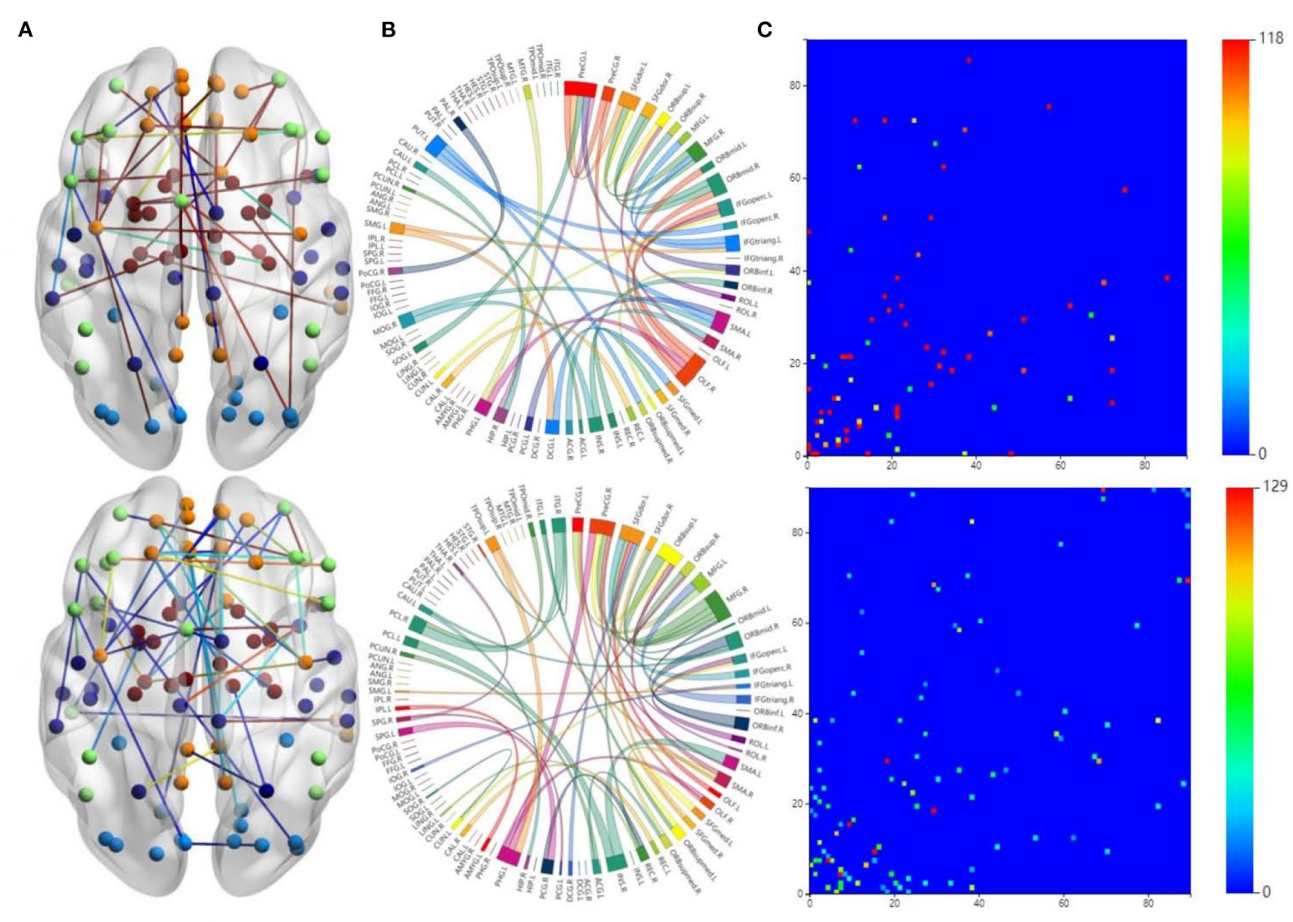
Visualizations of the connectivity of HC and neuropsychiatric disorders (NDs) in different manners on the CNP dataset. The first row and second row show the HC group and ND group, respectively. **(A)** Shows the connectivity in glass brain plots. **(B)** Shows the connectivity in circle plots. **(C)** Shows the connectivity in symmetric matrices.

The first row in Figure 3 is for the HC group, whereas the second row is for the ND group. Figure 3A shows 3D plots of the brain network to visualize the selected edges. A sphere denotes the center of a node. Different colors denote different brain regions. If two brain regions are functionally related, they are connected by a colored line. The colors of the lines indicate the edge strength and whether there is a positive correlation between the behaviors and the FCs. The brain network visualization has a small number of edges, which demonstrate the degree of the distribution across the whole brain network.

The 2D circle plots in Figure 3B are also used to visualize relationships between pairs of brain regions. The wider the edge between two regions, the closer their relationship is. These circle plots indicate how many FCs a region has with other brain regions.

Figure 3C has mappings of the 90 × 90 connectivity matrices, which are used to visualize aggregate statistics within and between predefined regions or networks. In a connectivity matrix, nodes represent brain regions and links measure conditional dependence between the brain regions. Brain connectivity analysis is equivalently transformed into the estimation of a spatial partial correlation matrix.

### 4.5. Analysis of HCs and NDs

In both HC group (the first row in Figure 3) and ND group (the second row in Figure 3), most of the FCs are common to both groups. These overlapping FCs are mainly within or across the temporal lobes or across the frontal, occipital, and parietal lobes, which confirm the results of previous studies. For instance, Haier et al. (2005) and Rubia et al. (2007) showed that temporal lobe dysfunction is strongly correlated with ADHD. Several brain regions in the frontal, parietal, temporal, and occipital lobes have been identified as significant predictors of ND (Gaudio et al., 2019; Zhang et al., 2020).

Furthermore, Figures 3A,B show that there are significant differences between the FCs of the two groups. Compared with the HCs, the ND group has abnormal brain regions, mainly in the supramarginal gyrus, cingulate gyrus, middle frontal gyrus, etc. Other studies have also found that there are fewer FCs in the middle frontal gyrus and anterior cingulate regions in SZ brains compared to HCs (Camchong et al., 2011; Liu et al., 2011). However, the FCs in the ND group are more complicated than those in the HC group, which may be due to their mental illness. These differences may affect the behaviors and mental states of the ND group. There are many highlighted cells in the HC matrix in Figure 3C, whereas the highlighted cells in the ND matrix are more dispersed. This also indicates that NDs may affect the FCs between brain regions.

### 4.6. Analysis of Different NDs

**Figure 4:**
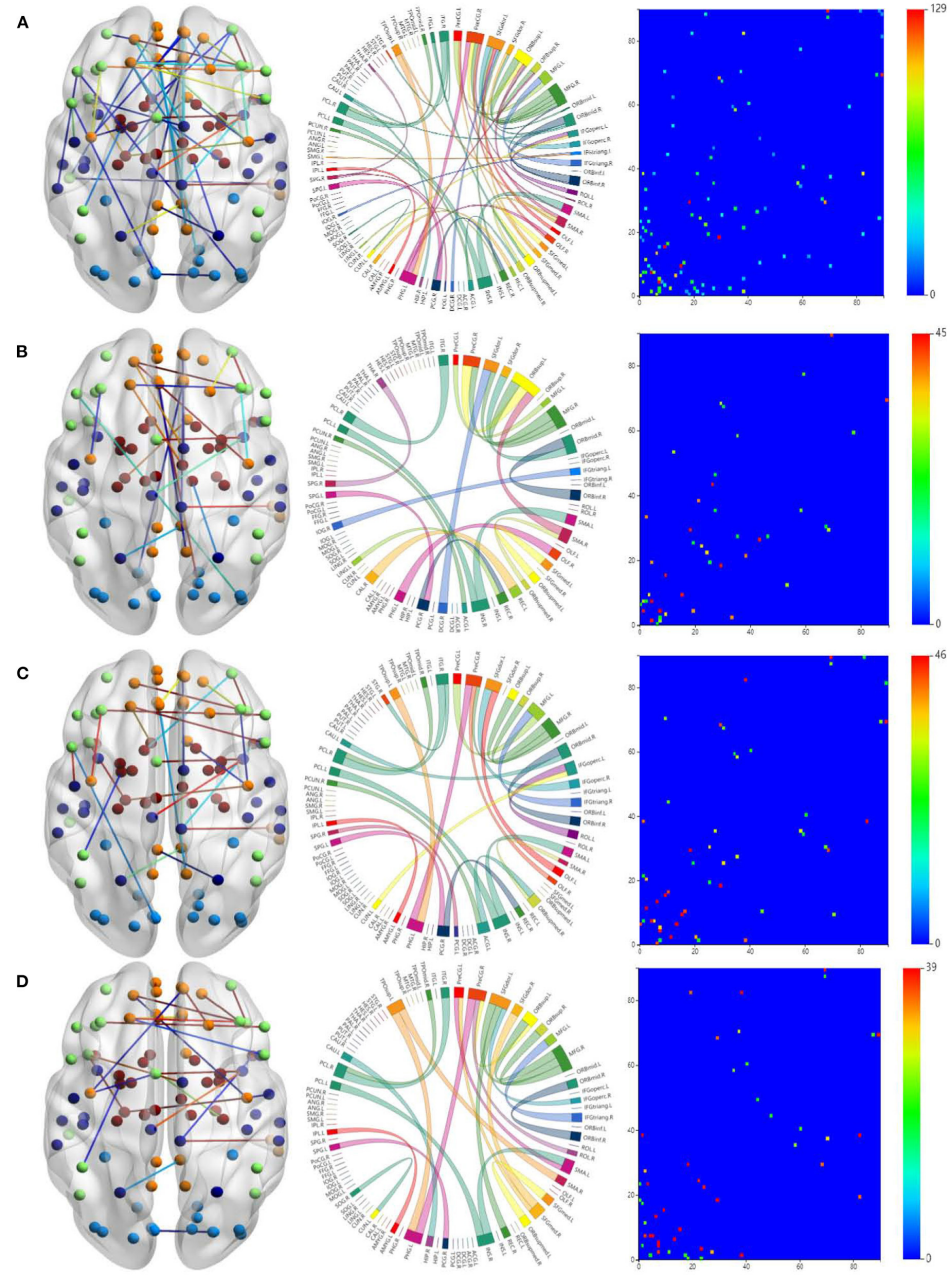
Visualizations of the connectivity of three ND subtypes in glass brain plot graph, circle plot graph, and symmetric matrix graph on CNP dataset. **(A)** Shows all the ND subtypes. **(B)** Shows the SZ subtype. **(C,D)** Show the BD and ADHD subtypes, respectively.

To study the specificity of subtypes in NDs, we visualized the FCs of the three ND subtypes in Figure 4. Figure 4A is for all the ND subtypes. Figure 4B is for the SZ subtype. Figures 4C,D are for the BD and ADHD subtypes, respectively.

The brain networks clearly suggest that the FCs of these diseases are very similar, but their differences are also very obvious. In particular, the FCs in the ADHD plots are obviously different from those in the SZ and BD plots. This is why classifying ADHD is usually a separate task in most approaches to classifying NDs. Moreover, the connections between brain regions shown in the circle plots in the second column are obviously different for the three diseases.

### 4.7. Features Distribution of PCA and DCL

**Figure 5:**
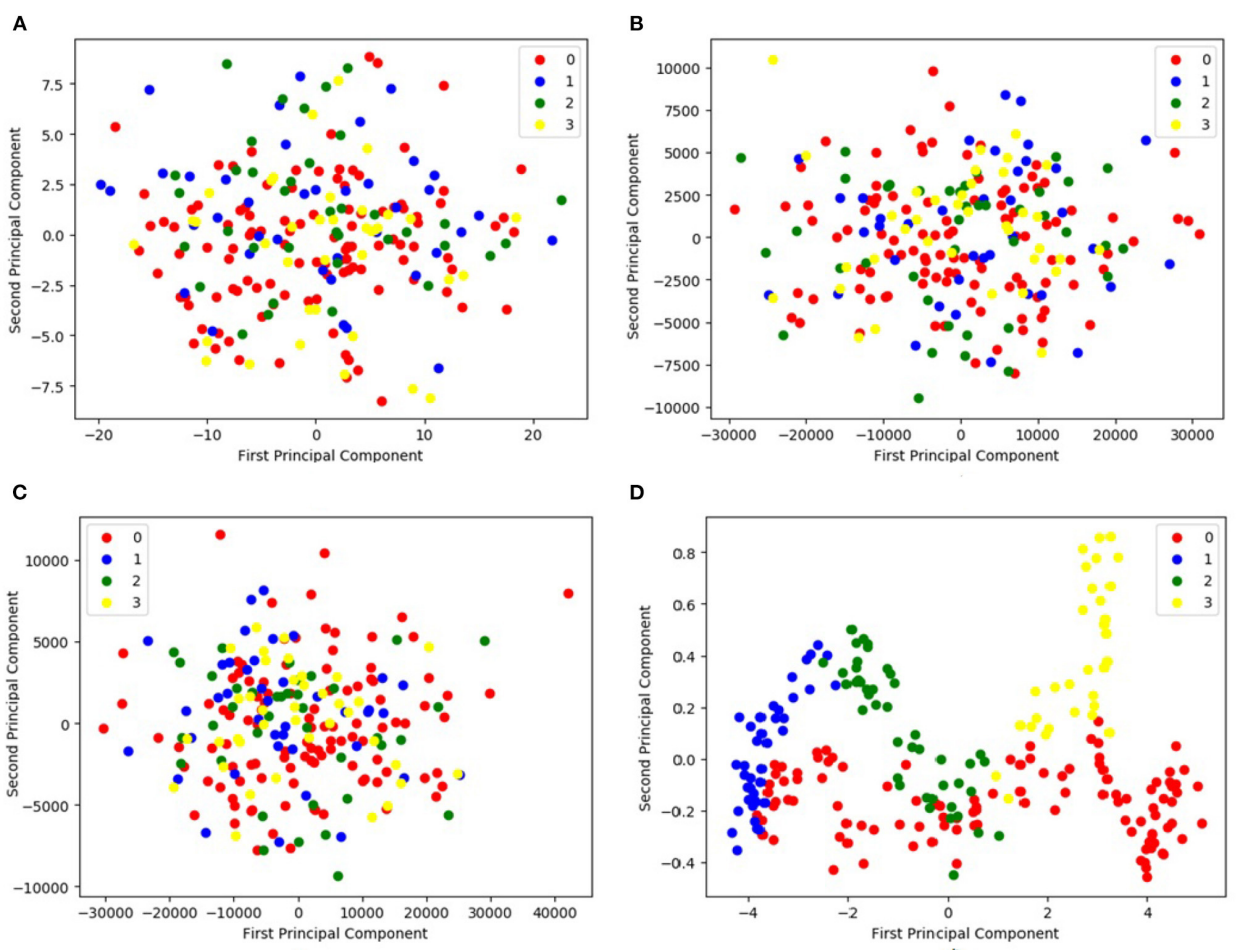
Representation of feature distribution on CNP dataset. **(A,B)** Visualize the fMRI and sMRI feature matrices processed by PCA, respectively. **(C)** Visualizes the combined feature matrix for the fMRI and sMRI images processed by PCA. **(D)** Visualizes the feature matrix produced by DCL. In the legend, 0 represents HC, 1 represents SZ, 2 represents BD, and 3 represents ADHD.

Figure 5 compares the principal components found by the PCA method with those found by the proposed DCL method. Figures 5A,B visualize the fMRI and sMRI feature matrices found by PCA. Figure 5C is the visualization of the combined feature matrix for the fMRI and sMRI images for PCA. Figure 5D is the feature matrix produced by DCL.

As shown in Figure 5, the figure shows that the three distributions of features produced by PCA are disordered (Figures 5A–C). Although the distributions of the PCA-processed fMRI and sMRI matrices (Figure 5C) are relatively concentrated, the four icons of subtypes are still indistinguishable. It would be difficult for classifiers to distinguish the features of the four subtypes. In contrast, the distribution of fMRI and sMRI matrices after DCL processing shows the effect of aggregation, which is shown in Figure 5D. The features of the four subtypes can be clearly distinguished. Therefore, the performance of a classifier would be greatly improved by using a feature matrix produced by the DCL method. At the same time, in order to eliminate the difference in the distribution of subtypes, we normalized the matrices in the DCL method, so that the subtypes are distributed in a smaller range.

## 5. Ablation Experiments and Discussion

We proposed the DCL framework to classify psychiatric disorders using fMRI and sMRI. In this section, we discussed several factors that influence the experimental results. To validate the performance of DCL on different size of datasets, we extended experiments on a larger sample size dataset (a subset of ADNI) and a small sample size dataset (a subset of OpenfMRI), respectively.

### 5.1. Influence of S-POET

The shrinkage principal orthogonal complement thresholding method is a covariance estimator with the approximate factor model, which is based on sparse PCA. In our method, we used the S-POET method to obtain asymptotic first-order distribution for the eigenvalues and eigenvectors of the fMRI and sMRI correlation matrices, respectively. To verify the effect of the S-POET method in our proposed DCL method, we extended two different DCL methods on XGBoost: one is based on PCA[DCL(PCA)] and another is based on S-POET[DCL(S-POET)].

As shown in Table 3, we extended the experiments on CNP dataset. For both datasets, compared with the DCL(PCA)-based XGBoost, the DCL(S-POET)-based XGBoost obtained the super performance. The accuracy was almost improved by 13% on CNP. Although S-POET is obtained by sparse PCA extension, S-POET is more suitable for sparse high-latitude data. PCA has widely been proved that it is a powerful tool for dimensionality reduction and data visualization. Its theoretical properties such as the consistency and asymptotic distributions of empirical eigenvalues and eigenvectors are challenging especially in the high dimensional regime. While, in the method S-POET, the spike magnitude of leading eigenvalues, sample size, and dimensionality of the leading eigenvalues are considered. In addition, a new covariance estimator is introduced in S-POET to correct the bias of PCA estimation of leading eigenvalues and eigenvectors. Therefore, S-POET is more advantageous in the process of fMRI and sMRI matrices analysis with high dimensionality and sparse features (Fan and Wang, 2015). Therefore, in the end, we build the DCL method with S-POET.

**Table 3:** Influence of shrinkage principal orthogonal complement thresholding method (S-POET) on XGBoost with CNP dataset.

**Method**	**ACC (%)**	**PRE (%)**	**REC (%)**	***F*_1_ (%)**
DCL(PCA)	59.00 (5.00)	60.00 (9.00)	61.00 (11.00)	59.00 (7.00)
DCL(S-POET)	72.00 (8.00)	81.00 (2.00)	70.00 (3.00)	75.00 (3.00)

### 5.2. Effectiveness of Different Inputs on XGBoosts

To verify the influence of different MRI modalities on model, we separately used fMRI, sMRI, and fMRI+sMRI matrices as inputs to three types of XGBoosts, namely PCAXGBoost, CCA-XGBoost, and DCL-XGBoost.

The results are shown in Table 4. The classification results of three XGBoost-based methods, using a single fMRI or sMRI matrix as input, are similar. However, the results of using PCA, CCA, and DCL processed fMRI and sMRI matrices as input to the XGBoost classifier have greatly improved. Especially for the DCL-XGBoost method, the accuracy is improved by almost 14% on the CNP dataset. As the two modalities complement each other, their combination results in higher classification accuracy. Furthermore, the performance of PCA and CCA-processed matrices is not as good as when using DCL-processed matrices as the XGBoost input.

**Table 4:** Evaluation of different inputs to the different combinations of XGBoost on the CNP dataset.

**Method**	**Input**	**ACC (%)**	**PRE (%)**	**REC (%)**	***F*_1_ (%)**
PCA+XGBoost	fMRI	38.00 (7.00)	35.00 (4.00)	36.16 (3.00)	33.00 (2.00)
	sMRI	37.00 (8.00)	35.00 (3.00)	36.00 (10.00)	32.00 (9.00)
	fMRI+sMRI	49.00 (11.00)	45.00 (8.00)	49.00 (8.00)	45.00 (7.00)
CCA+XGBoost	fMRI	36.00 (10.00)	34.00 (4.00)	36.00 (7.00)	35.00 (8.00)
	sMRI	38.20 (1.00)	37.06 (7.00)	35.00 (9.00)	36.00 (4.00)
	fMRI+sMRI	49.00 (8.00)	46.00 (14.00)	49.00 (12.00)	44.00 (14.00)
DCL-XGBoost	fMRI	56.00 (2.00)	58.00 (8.00)	60.00 (3.00)	53.00 (9.00)
	sMRI	58.00 (6.00)	62.00 (8.00)	52.00 (11.00)	55.00 (6.00)
	fMRI+sMRI	72.00 (8.00)	81.00 (2.00)	70.00 (3.00)	75.00 (3.00)

### 5.3. Influence of Medication Taken

**Figure 6:**
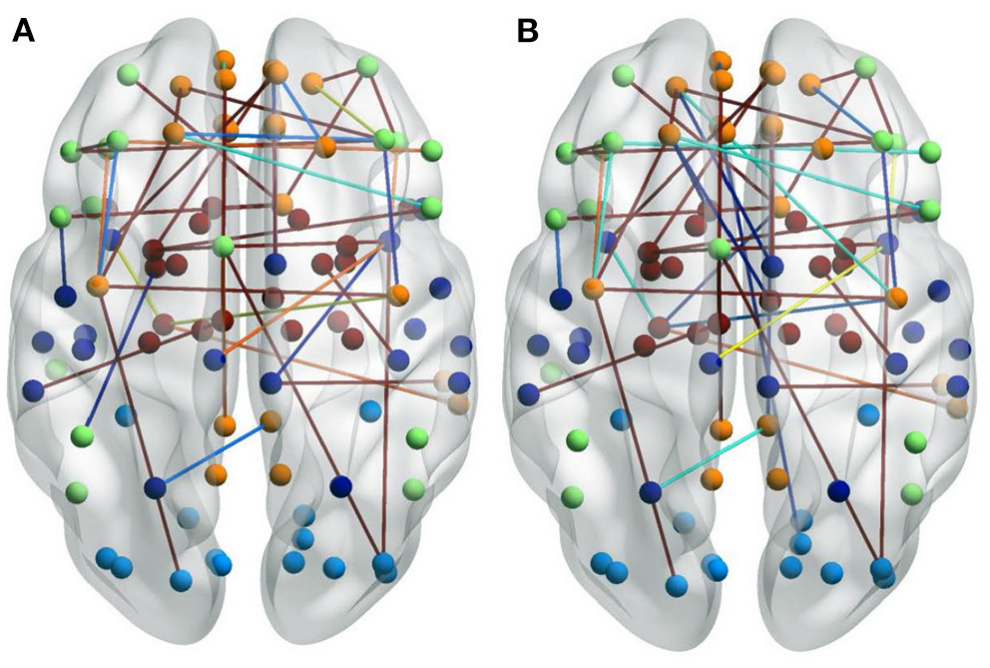
Visualizations of the connectivity of NDs who took medicine or not in the glass brain plot graph on the CNP dataset. **(A)** Shows NDs without medication. **(B)** Shows NDs with medication.

Some patients in the ND group had taken medication for their mental illness. To analyze the impact of these medications on the patients, we visualized the selected FCs for a group who had taken medication and for a group who had not. There are significant differences between these two groups, as shown in Figure 6. Figure 6A shows NDs without medication. Figure 6B shows NDs with medication. The representations of the FCs over the whole brain are similar, but for the group who had not used medication, there are more edges over the boundary of the brain. This may be due to the fact that some FCs are interrupted by the patient taking certain medication, resulting in remission or deepening of mental illness.

### 5.4. Extend Experiments

To verify the performance of DCL on different datasets, we extended experiments on a larger sample size dataset (a subset of ADNI) and a small sample size dataset (a subset of OpenfMRI), respectively. The Alzheimer's Disease Neuroimaging Initiative (ADNI) (Carrillo et al., 2012) is a large dataset including Alzheimer's disease (AD) and mild cognitive impairment (MCI). We selected a subset of the ADNI dataset to evaluate our proposed DCL method. This subset includes 420 samples with sMRI (T1w MRI) and fMRI (rs-fMRI). It consists of 105 subjects with AD, 105 late mild cognitive impairment (LMCI) subjects, 105 early mild cognitive impairment (EMCI) subjects, and 105 HC subjects. The OpenfMRI Poldrack et al. (2013) was designed to serve as a repository for the open sharing and dissemination of task-based fMRI data. As it has grown, it has broadened to encompass other data types as well, including EEG, MEG, rs-fMRI (fMRI), and diffusion MRI (sMRI), which were acquired on both healthy and clinical populations. We selected a small subset of OpenfMRI dataset with the resting state. This subset includes 93 samples with sMRI and fMRI. It consists of 20 HC subjects, 16 BD subjects, 28 SC subjects, and 29 ADHD subjects.

In our study, the subsets of ADNI and OpenfMRI are used as the external datasets to evaluate the performance of DCL. The data processing steps followed the manner in Section 3. The experimental design and metrics follow the design in Section 4. The classification results of this subset are show in Table 5. We also used three typical machine learning methods (SVM, RF, and XGBoost) as the baseline. As shown in Tables 5, 6, the accuracy trend of the experimental results is similar to that in Table 2. The DCL-based classifiers achieve much better classification results, which further proves that the DCL method can reduce the complexity of the data by preprocessing the two types of MRI, thereby improving the classification performance of the classifiers. By comparing Tables 2, 5, 6, it can be found that the classification results of the three classifiers on the subset of ADNI achieve the best performance and that on the subset of OpenfMRI achieve the worst performance. In addition to the reasons for the samples themselves, in these three datasets, the subset of ADNI has the largest sample size, which can lead to better training and prediction of the machine learning methods. While the subset of OpenfMRI has the smallest sample size, which limits the training and prediction of the machine learning methods. Furthermore, in the case of a limited sample size on the subset of OpenfMRI, the performance of DCL-based methods got obvious advantages compared to other methods.

**Table 5:** Mean values in the evaluation of the classification performance on the subset of Alzheimer's Disease Neuroimaging Initiative (ADNI).

**Classifier**	**ACC (%)**	**PRE (%)**	**REC (%)**	***F*_1_ (%)**
SVM	54.00 (8.00)	53.00 (5.00)	59.00 (4.00)	57.00 (7.00)
RF	54.00 (4.00)	52.00 (10.00)	58.00 (4.00)	55.00 (8.00)
XGBoost	55.00 (10.00)	52.00 (15.00)	58.00 (7.00)	56.00 (9.00)
PCA+SVM	60.00 (10.00)	62.00 (4.00)	61.00 (7.00)	60.00 (6.00)
PCA+RF	65.00 (7.00)	61.00 (8.00)	63.00 (10.00)	63.00 (3.00)
PCA+XGBoost	72.00 (3.00)	68.00 (10.00)	73.00 (13.00)	72.00 (9.00)
CCA+SVM	62.00 (10.00)	63.00 (11.00)	65.00 (8.00)	63.00 (7.00)
CCA+RF	62.00 (4.00)	64.00 (3.00)	66.00 (6.00)	62.00 (6.00)
CCA+XGBoost	75.00 (4.00)	73.00 (6.00)	76.00 (7.00)	75.00 (9.00)
DCL+SVM	77.00 (12.00)	78.00 (3.00)	77.00 (9.00)	79.00 (10.00)
DCL+RF	78.00 (6.00)	79.00 (4.00)	78.00 (10.00)	80.00 (13.00)
DCL+XGBoost	80.00 (9.00)	79.00 (9.00)	80.00 (5.00)	82.00 (7.00)

**Table 6:** Mean values in the evaluation of the classification performance on the subset of OpenfMRI.

**Classifier**	**ACC (%)**	**PRE (%)**	**REC (%)**	***F*_1_ (%)**
SVM	33.00 (7.00)	33.00 (3.00)	35.00 (9.00)	34.00 (8.00)
RF	34.00 (6.00)	34.00 (11.00)	33.00 (2.00)	35.00 (7.00)
XGBoost	35.00 (6.00)	35.00 (7.00)	34.00 (9.00)	36.00 (2.00)
PCA+SVM	39.00 (7.00)	38.00 (10.00)	39.00 (10.00)	40.00 (4.00)
PCA+RF	41.00 (2.00)	40.00 (10.00)	41.00 (6.00)	41.00 (13.00)
PCA+XGBoost	43.00 (9.00)	44.00 (8.00)	45.00 (8.00)	42.00 (7.00)
CCA+SVM	43.00 (2.00)	44.00 (5.00)	46.00 (9.00)	45.00 (2.00)
CCA+RF	45.00 (7.00)	46.00 (7.00)	47.00 (3.00)	46.00 (10.00)
CCA+XGBoost	51.00 (14.00)	53.00 (7.00)	56.00 (7.00)	50.00 (5.00)
DCL+SVM	55.00 (6.00)	57.00 (8.00)	56.00 (8.00)	57.00 (6.00)
DCL+RF	62.00 (10.00)	64.00 (3.00)	64.00 (7.00)	63.00 (9.00)
DCL+XGBoost	67.00 (8.00)	69.00 (10.00)	68.00 (9.00)	68.00 (10.00)

We compared DCL+XGBoost with several other well-established methods that were designed for the multi neuropsychiatric disorders classification: mMLDA (Janousova et al., 2015), MFMK-SVM (Liu J. et al., 2018), KFCM (Baskar et al., 2019), MK-SVM (Zhuang et al., 2019), and mRMR-SVM (Zhang et al., 2021). These methods used one or both types of MRI data as input of the model for multi neuropsychiatric disorder classification. These methods were trained using different datasets and utilize very different predictive architectures. We either re-implemented them exactly as described by the authors or used the code released by the author. To ensure that the comparative evaluation is fair, we used the same training data and test data for all considered methods on tree datasets. The results are shown in Table 7, it can be found that our proposed method achieves state-of-the-art performance on all three datasets. These methods needed much more feature selection work and parameter settings, for example, mRMR-SVM needs mutual selected information as a measure to solve the trade-off between feature redundancy and relevance (Morgado et al., 2015). It increases the difficulty of model optimization. In addition, the performance of these methods improved as the sample size increased. This means that sample size and model performance are positively correlated.

**Table 7:** Comparison results with other methods on tree datasets.

**Dataset**	**Classifier**	**MRIs**	**ACC (%)**	**PRE (%)**	**REC (%)**	***F*_1_ (%)**
CNP	mMLDA (Janousova et al., 2015)	sMRI	65.00 (7.00)	65.00 (6.00)	67.00 (9.00)	64.00 (6.00)
	MFMK-SVM (Liu J. et al., 2018)	sMRI, DTI	67.00 (9.00)	64.00 (12.00)	65.00 (7.00)	68.00 (9.00)
	KFCM (Baskar et al., 2019)	sMRI	70.00 (7.00)	71.00 (7.00)	70.00 (6.00)	69.00 (10.00)
	MK-SVM (Zhuang et al., 2019)	sMRI, fMRI	70.00 (11.00)	75.00 (4.00)	72.00 (4.00)	74.00 (7.00)
	mRMR-SVM (Zhang et al., 2021)	sMRI, fMRI	71.00 (9.00)	78.00 (7.00)	71.00 (6.00)	72.00 (10.00)
	DCL+XGBoost	sMRI, fMRI	72.00 (8.00)	81.00 (2.00)	70.00 (3.00)	75.00 (3.00)
ADNI	mMLDA (Janousova et al., 2015)	sMRI	70.00 (8.00)	72.00 (8.00)	70.00 (10.00)	69.00 (9.00)
	MFMK-SVM (Liu J. et al., 2018)	sMRI, DTI	73.00 (9.00)	72.00 (10.00)	74.00 (6.00)	75.00 (7.00)
	KFCM (Baskar et al., 2019)	sMRI	75.00 (9.00)	74.00 (1.00)	76.00 (4.00)	74.00 (8.00)
	MK-SVM (Zhuang et al., 2019)	sMRI, fMRI	75.00 (11.00)	74.00 (9.00)	75.00 (8.00)	75.00 (2.00)
	mRMR-SVM (Zhang et al., 2021)	sMRI, fMRI	79.00 (12.00)	82.00 (10.00)	79.00 (6.00)	81.00 (7.00)
	DCL+XGBoost	sMRI, fMRI	80.00 (9.00)	79.00 (9.00)	80.00 (5.00)	82.00 (7.00)
OpenfMRI	mMLDA (Janousova et al., 2015)	sMRI	54.00 (7.00)	53.00 (10.00)	55.00 (7.00)	53.00 (9.00)
	MFMK-SVM (Liu J. et al., 2018)	sMRI, DTI	57.00 (5.00)	58.00 (7.00)	56.00 (10.00)	57.00 (6.00)
	KFCM (Baskar et al., 2019)	sMRI	63.00 (11.00)	64.00 (8.00)	64.00 (4.00)	64.00 (9.00)
	MK-SVM (Zhuang et al., 2019)	sMRI, fMRI	66.00 (8.00)	65.00 (12.00)	67.00 (8.00)	64.00 (10.00)
	mRMR-SVM (Zhang et al., 2021)	sMRI, fMRI	67.00 (5.00)	70.00 (7.00)	71.00 (6.00)	72.00 (10.00)
	DCL+XGBoost	sMRI, fMRI	67.00 (8.00)	69.00 (10.00)	68.00 (9.00)	68.00 (10.00)

### 5.5. Limitations

There are several limitations to this study. (1) We used only MRI data as the input. However, the classification of complex disorders could be made more accurate by including phenotypic information. (2) The amount and uneven quality of the MRI data have a significant influence on the performance of a model and reduce the accuracy of classification.

## 6. Conclusion

This work demonstrated that the DCL method can effectively combine different information from fMRI and sMRI images. DCL identifies both the common and distinct information between the two input MRI matrices. The decomposition-based CCA is used to analyze the correlation and construct the required matrices. Thus, DCL has better performance in both classification and identifying FCs. The DCL method can be used to detect complex and nonlinear relationships between the two types of MRI images. Our experiments showed that the DCL method can improve classification performance so that it is a suitable method for classifying mental illnesses.

## Data Availability Statement

The original contributions presented in the study are included in the article/supplementary material, further inquiries can be directed to the corresponding author/s.

## Author Contributions

LL, HZ, and GL contributed to the conception and design of the study. JC organized the database. YW performed the statistical analysis. LL wrote the first draft of the manuscript. Y-PW modified the manuscript. All authors contributed to manuscript revision, read, and approved the submitted version.

## Funding

This work was funded partially by the National Natural Science Foundation of China under Grant Nos. 62172444, 61877059, and 62102454, the 111 Project (No. B18059), and the Henan Provincial Key Research and Promotion Projects (No. 222102310085).

## Conflict of Interest

The authors declare that the research was conducted in the absence of any commercial or financial relationships that could be construed as a potential conflict of interest.

## Publisher's Note

All claims expressed in this article are solely those of the authors and do not necessarily represent those of their affiliated organizations, or those of the publisher, the editors and the reviewers. Any product that may be evaluated in this article, or claim that may be made by its manufacturer, is not guaranteed or endorsed by the publisher.

## References

[B1] Baskar D. Jayanthi V. Jayanthi A. (2019). An efficient classification approach for detection of Alzheimer's disease from biomedical imaging modalities. Multim. Tools Appl. 78, 12883–12915. 10.1007/s11042-018-6287-8

[B2] Calhoun V. D. Adali T. Kiehl K. A. Astur R. Pearlson G. D. (2010). A method for multitask fMRI data fusion applied to schizophrenia. Hum. Brain Mapp. 27, 598–610. 10.1002/hbm.20204 16342150 PMC2751648

[B3] Camchong J. MacDonald A. W. III Bell C. Mueller B. A. Lim K. O. (2011). Altered functional and anatomical connectivity in schizophrenia. Schizophr. Bull. 37, 640–650. 10.1093/schbul/sbp131 19920062 PMC3080691

[B4] Carrillo M. C. Bain L. J. Frisoni G. B. Weiner M. W. (2012). Worldwide Alzheimer's disease neuroimaging initiative. Alzheimer's Dement. 8, 337–342. 10.1016/j.jalz.2012.04.007 22748939

[B5] Connaughton M. Whelan R. O'Hanlon E. McGrath J. (2022). White matter microstructure in children and adolescents with ADHD. NeuroImage 2022, 102957. 10.1016/j.nicl.2022.102957 35149304 PMC8842077

[B6] Correa N. M. Eichele T. Adali T. Li Y. O. Calhoun V. D. (2010). Multi-set canonical correlation analysis for the fusion of concurrent single trial ERP and functional MRI. Neuroimage 50, 1438–1445. 10.1016/j.neuroimage.2010.01.062 20100584 PMC2857695

[B7] de Filippis R. Carbone E. A. Gaetano R. Bruni A. Pugliese V. Segura-Garcia C.. (2019). Machine learning techniques in a structural and functional MRI diagnostic approach in schizophrenia: a systematic review. Neuropsychiatr. Dis. Treat. 15, 1605. 10.2147/NDT.S202418 31354276 PMC6590624

[B8] Dubois J. Adolphs R. (2016). Building a science of individual differences from fMRI. Trends Cogn. Sci. 20, 425–443. 10.1016/j.tics.2016.03.014 27138646 PMC4886721

[B9] Fan J. Wang W. (2015). Asymptotics of empirical Eigen-structure for ultra-high dimensional spiked covariance model. arXiv[Preprint].arXiv:1502.04733.

[B10] Fan Z. Xu F. Qi X. Li C. Yao L. (2020). Classification of Alzheimer disease based on brain MRI and machine learning. Neural Comput. Appl. 32, 1927–1936. 10.1007/s00521-019-04495-0

[B11] Finn E. S. Shen X. Scheinost D. Rosenberg M. D. Huang J. Chun M. M.. (2015). Functional connectome fingerprinting: identifying individuals using patterns of brain connectivity. Nat. Neurosci. 18, 1664–1671. 10.1038/nn.4135 26457551 PMC5008686

[B12] Gao S. Calhoun V. D. Sui J. (2020). Multi-modal component subspace-similarity-based multi-kernel SVM for schizophrenia classification, in Medical Imaging 2020: Computer-Aided Diagnosis: International Society for Optics and Photonics 113143X (Houston, TX: SPIE Medical Imaging). 10.1117/12.2550339

[B13] Gaudio S. Carducci F. Piervincenzi C. Olivo G. Schioth H. B. (2019). Altered thalamo-cortical and occipital-parietal-temporal-frontal white matter connections in patients with anorexia and bulimia nervosa: a systematic review of diffusion tensor imaging studies. J. Psychiatry Neurosci. 44, 324–339. 10.1503/jpn.180121 30994310 PMC6710091

[B14] Gorgolewski K. J. Auer T. Calhoun V. D. Craddock R. C. Das S. Duff E. P.. (2016). The brain imaging data structure, a format for organizing and describing outputs of neuroimaging experiments. Sci. Data 3, 160044–160044. 10.1038/sdata.2016.44 27326542 PMC4978148

[B15] Gorgolewski K. J. Durnez J. Poldrack R. A. (2017). Preprocessed consortium for neuropsychiatric phenomics dataset. F1000Research 6, 1262. 10.12688/f1000research.11964.1 29152222 PMC5664981

[B16] Groves A. R. Beckmann C. F. Smith S. M. Woolrich M. W. (2011). Linked independent component analysis for multimodal data fusion. Neuroimage 54, 2198–2217. 10.1016/j.neuroimage.2010.09.073 20932919

[B17] Haier R. J. Jung R. E. Yeo R. A. Head K. Alkire M. T. (2005). The neuroanatomy of general intelligence: sex matters. NeuroImage 25, 320–327. 10.1016/j.neuroimage.2004.11.019 15734366

[B18] Han K.-M. De Berardis D. Fornaro M. Kim Y.-K. (2019). Differentiating between bipolar and unipolar depression in functional and structural MRI studies. Prog. Neuro Psychopharmacol. Biol. Psychiatry 91, 20–27. 10.1016/j.pnpbp.2018.03.022 29601896

[B19] Heinrichs R. W. Zakzanis K. K. (1998). Neurocognitive deficit in schizophrenia: a quantitative review of the evidence. Neuropsychology 12, 426. 10.1037/0894-4105.12.3.426 9673998

[B20] Hu W. Cai B. Zhang A. Calhoun V. D. Wang Y.-P. (2019). Deep collaborative learning with application to the study of multimodal brain development. IEEE Trans. Biomed. Eng. 66, 3346–3359. 10.1109/TBME.2019.2904301 30872216 PMC8177041

[B21] Hu W. Meng X. Bai Y. Zhang A. Qu G. Cai B.. (2021). Interpretable multimodal fusion networks reveal mechanisms of brain cognition. IEEE Trans. Med. Imaging 40, 1474–1483. 10.1109/TMI.2021.3057635 33556002 PMC8208525

[B22] Janousova E. Schwarz D. Kasparek T. (2015). Combining various types of classifiers and features extracted from magnetic resonance imaging data in schizophrenia recognition. Psychiatry Res. 232, 237–249. 10.1016/j.pscychresns.2015.03.004 25912090

[B23] Jiang R. Calhoun V. D. Cui Y. Qi S. Zhuo C. Li J.. (2020). Multimodal data revealed different neurobiological correlates of intelligence between males and females. Brain Imaging Behav. 14, 1979–1993. 10.1007/s11682-019-00146-z 31278651 PMC7416494

[B24] Jiang Y. Duan M. He H. Yao D. Luo C. (2021). Structural and functional MRI brain changes in patients with schizophrenia following electroconvulsive therapy: a systematic review. Curr. Neuropharmacol. 10.2174/1570159X19666210809101248 34370638 PMC9886826

[B25] Joshi A. Scheinost D. Okuda H. Belhachemi D. Murphy I. Staib L. H.. (2011). Unified framework for development, deployment and robust testing of neuroimaging algorithms. Neuroinformatics 9, 69–84. 10.1007/s12021-010-9092-8 21249532 PMC3066099

[B26] Kessler R. C. Petukhova M. Sampson N. A. Zaslavsky A. M. Wittchen H. (2012). Twelve-month and lifetime prevalence and lifetime morbid risk of anxiety and mood disorders in the United States. Int. J. Methods Psychiatr. Res. 21, 169–184. 10.1002/mpr.1359 22865617 PMC4005415

[B27] Lake E. M. Finn E. S. Noble S. M. Vanderwal T. Shen X. Rosenberg M. D.. (2019). The functional brain organization of an individual allows prediction of measures of social abilities trans-diagnostically in autism and attention/deficit and hyperactivity disorder. Biol. Psychiatry 86, 315–326. 10.1016/j.biopsych.2019.02.019 31010580 PMC7311928

[B28] Liu H. Fan G. Xu K. Wang F. (2011). Changes in cerebellar functional connectivity and anatomical connectivity in schizophrenia: a combined resting-state functional MRI and diffusion tensor imaging study. J. Magn. Reson. Imaging 34, 1430–1438. 10.1002/jmri.22784 21976249 PMC3221764

[B29] Liu J. Demirci O. Calhoun V. D. (2008). A parallel independent component analysis approach to investigate genomic influence on brain function. IEEE Signal Process.Lett. 15, 413–416. 10.1109/LSP.2008.922513 19834575 PMC2761666

[B30] Liu J. Wang X. Zhang X. Pan Y. Wang X. Wang J. (2018). MMM: classification of schizophrenia using multi-modality multi-atlas feature representation and multi-kernel learning. Multim. Tools Appl. 77, 29651–29667. 10.1007/s11042-017-5470-7

[B31] Liu L. Chen S. Zhu X. Zhao X.-M. Wang J. (2019). Deep convolutional neural network for accurate segmentation and quantification of white matter hyperintensities. Neurocomputing 384, 231–242. 10.1016/j.neucom.2019.12.05033574101

[B32] Liu Z. Zhang J. Xie X. Rolls E. T. Sun J. Zhang K.. (2018). Neural and genetic determinants of creativity. Neuroimage 174, 164–167. 10.1016/j.neuroimage.2018.02.067 29518564

[B33] Madeira N. Duarte J. V. Martins R. Costa G. N. Macedo A. Castelo-Branco M. (2020). Morphometry and gyrification in bipolar disorder and schizophrenia: a comparative MRI study. NeuroImage 26, 102220. 10.1016/j.nicl.2020.102220 32146321 PMC7063231

[B34] McIntosh A. M. Job D. E. Moorhead T. W. J. Harrison L. K. Lawrie S. M. Johnstone E. C. (2005). White matter density in patients with schizophrenia, bipolar disorder and their unaffected relatives. Biol. Psychiatry 58, 254–257. 10.1016/j.biopsych.2005.03.044 15939409

[B35] Mill R. D. Winfield E. C. Cole M. W. Ray S. (2021). Structural MRI and functional connectivity features predict current clinical status and persistence behavior in prescription opioid users. NeuroImage 30, 102663. 10.1016/j.nicl.2021.102663 33866300 PMC8060550

[B36] Morgado P. M. Silveira M. for the Alzheimer's Disease Neuroimaging Initiative (2015). Minimal neighborhood redundancy maximal relevance: application to the diagnosis of Alzheimer' s disease. Neurocomputing 155, 295–308. 10.1016/j.neucom.2014.12.070

[B37] Olesen P. J. Nagy Z. Westerberg H. Klingberg T. (2003). Combined analysis of DTI and fMRI data reveals a joint maturation of white and grey matter in a fronto-parietal network. Cogn. Brain Res. 18, 48–57. 10.1016/j.cogbrainres.2003.09.003 14659496

[B38] Poldrack R. A. Barch D. M. Mitchell J. Wager T. Wagner A. D. Devlin J. T.. (2013). Toward open sharing of task-based fMRI data: the openfMRI project. Front. Neuroinformatics 7, 12. 10.3389/fninf.2013.00012 23847528 PMC3703526

[B39] Poldrack R. A. Congdon E. Triplett W. Gorgolewski K. J. Karlsgodt K. H. Mumford J. A.. (2016). A phenome-wide examination of neural and cognitive function. Sci. Data 3, 160110. 10.1038/sdata.2016.110 27922632 PMC5139672

[B40] Qiao C. Lu L. Yang L. Kennedy P. J. (2019). Identifying brain abnormalities with schizophrenia based on a hybrid feature selection technology. Appl. Sci. 9, 2148. 10.3390/app9102148

[B41] Rakić M. Cabezas M. Kushibar K. Oliver A. Llado X. (2020). Improving the detection of autism spectrum disorder by combining structural and functional MRI information. NeuroImage 25, 102181. 10.1016/j.nicl.2020.102181 31982680 PMC6994708

[B42] Rosenberg M. D. Casey B. J. Holmes A. J. (2018). Prediction complements explanation in understanding the developing brain. Nat. Commun. 9, 589. 10.1038/s41467-018-02887-9 29467408 PMC5821815

[B43] Rubia K. Smith A. B. Brammer M. J. Taylor E. (2007). Temporal lobe dysfunction in medication-nave boys with attention-deficit/hyperactivity disorder during attention allocation and its relation to response variability. Biol. Psychiatry 62, 999–1006. 10.1016/j.biopsych.2007.02.024 17585887

[B44] Seghier M. L. Lazeyras F. Zimine S. Maier S. E. Hanquinet S. Delavelle J.. (2004). Combination of event-related fMRI and diffusion tensor imaging in an infant with perinatal stroke. Neuroimage 21, 463–472. 10.1016/j.neuroimage.2003.09.015 14741684

[B45] Strasser H. C. Lilyestrom J. Ashby E. R. Honeycutt N. A. Schretlen D. J. Pulver A. E.. (2005). Hippocampal and ventricular volumes in psychotic and nonpsychotic bipolar patients compared with schizophrenia patients and community control subjects: a pilot study. Biol. Psychiatry 57, 633–639. 10.1016/j.biopsych.2004.12.009 15780850

[B46] Su C. Xu Z. Pathak J. Wang F. (2020). Deep learning in mental health outcome research: a scoping review. Transl. Psychiatry 10, 1–26. 10.1038/s41398-020-0780-3 32532967 PMC7293215

[B47] Sui J. Adali T. Pearlson G. D. Calhoun V. D. (2009). An ICA-based method for the identification of optimal fMRI features and components using combined group-discriminative techniques. Neuroimage 46, 73–86. 10.1016/j.neuroimage.2009.01.026 19457398 PMC4356027

[B48] Sui J. Pearlson G. Caprihan A. Adali T. Kiehl K. A. Liu J.. (2011). Discriminating schizophrenia and bipolar disorder by fusing fMRI and DTI in a multimodal CCA+ joint ICA model. Neuroimage 57, 839–855. 10.1016/j.neuroimage.2011.05.055 21640835 PMC3129373

[B49] Wang F. Kalmar J. H. He Y. Jackowski M. Chepenik L. G. Edmiston E. E.. (2009). Functional and structural connectivity between the perigenual anterior cingulate and amygdala in bipolar disorder. Biol. Psychiatry 66, 516–521. 10.1016/j.biopsych.2009.03.023 19427632 PMC2830492

[B50] Willemink M. J. Koszek W. A. Hardell C. Wu J. Fleischmann D. Harvey H.. (2020). Preparing medical imaging data for machine learning. Radiology 295, 4–15. 10.1148/radiol.2020192224 32068507 PMC7104701

[B51] Yu Y. Li M. Liu L. Li Y. Wang J. (2019). Clinical big data and deep learning: applications, challenges and future outlooks. Big Data Mining Analyt. 2, 288–305. 10.26599/BDMA.2019.9020007

[B52] Zhang L. Ai H. Opmeer E. M. Marsman J. C. Der Meer L. V. Ruhe H. G.. (2020). Distinct temporal brain dynamics in bipolar disorder and schizophrenia during emotion regulation. Psychol. Med. 50, 413–421. 10.1017/S0033291719000217 30773147 PMC7025159

[B53] Zhang T. Liao Q. Zhang D. Zhang C. Yan J. Ngetich R.. (2021). Predicting MCI to ad conversation using integrated sMRI and RS-fMRI: machine learning and graph theory approach. Front. Aging Neurosci. 13, 688926. 10.3389/fnagi.2021.688926 34421570 PMC8375594

[B54] Zhuang H. Liu R. Wu C. Meng Z. Wang D. Liu D.. (2019). Multimodal classification of drug-naïve first-episode schizophrenia combining anatomical, diffusion and resting state functional resonance imaging. Neurosci. Lett. 705, 87–93. 10.1016/j.neulet.2019.04.039 31022433

